# Comparison of Human Experts and AI in Predicting Autism from Facial Behavior

**Published:** 2023-03-16

**Authors:** Evangelos Sariyanidi, Casey J. Zampella, Ellis DeJardin, John D. Herrington, Robert T. Schultz, Birkan Tunc

**Affiliations:** 1 Center for Autism Research, The Children’s Hospital of Philadelphia, United States; 2 University of Pennsylvania, United States

**Keywords:** autism, assistive healthcare technologies, digital phenotyping

## Abstract

Advances in computational behavior analysis via artificial intelligence (AI) promise to improve mental healthcare services by providing clinicians with tools to assist diagnosis or measurement of treatment outcomes. This potential has spurred an increasing number of studies in which automated pipelines predict diagnoses of mental health conditions. However, a fundamental question remains unanswered: How do the predictions of the AI algorithms correspond and compare with the predictions of humans? This is a critical question if AI technology is to be used as an assistive tool, because the utility of an AI algorithm would be negligible if it provides little information beyond what clinicians can readily infer. In this paper, we compare the performance of 19 human raters (8 autism experts and 11 non-experts) and that of an AI algorithm in terms of predicting autism diagnosis from short (3-minute) videos of *N* = 42 participants in a naturalistic conversation. Results show that the AI algorithm achieves an average accuracy of 80.5%, which is comparable to that of clinicians with expertise in autism (83.1%) and clinical research staff without specialized expertise (78.3%). Critically, diagnoses that were inaccurately predicted by most humans (experts and non-experts, alike) were typically correctly predicted by AI. Our results highlight the potential of AI as an assistive tool that can augment clinician diagnostic decision-making.

## 1. Introduction

Modern medical disciplines typically rely on a variety of technological tools to assist in diagnosis and monitor treatment progress. From brain imaging technologies to blood and genetic tests, instruments that assist medical decision-makers are a cornerstone of modern medicine. In the domain of psychiatry and psychology, however, medical decision-making relies nearly exclusively on observational or paper-and-pencil instruments. Thus, recent advances in computer vision and artificial intelligence (AI) are poised to rapidly advance research and clinical decision-making in psychiatry by introducing reliable and granular tools within a new paradigm: computational behavior analysis [1, 2, 3, 4, 5]. Such tools can capture and quantify human behavior with extraordinary precision, even from brief video recordings.

Autism spectrum disorder (ASD), like nearly all psychiatric conditions, is defined by observable behavioral cues—what a person does well or not well, too little or too much. Its core traits include observable differences in social communication, social reciprocity, nonverbal communication, and relationships, as well as restricted patterns of interests and activities [6]. The current reliance on assessment and interpretation of overt behavior makes autism an excellent candidate for computational behavior analysis approaches. Coupling computationally-derived biomarkers with expert clinician judgment may provide an extremely potent approach to autism care, by enhancing the currently limited reliability of clinical assessments (*e.g.,* DSM-5 field trials Kappa = 0.69) [7], shortening lengthy diagnostic evaluations, and improving sensitivity for capturing change over the course of treatment and development.

This potential has spurred a plethora of studies that aim to diagnose autism via AI pipelines based on various behavioral modalities and sensors [8]. Notably, to our knowledge, no study has directly compared AI algorithms and human raters with respect to overall predictive capacity or specific decisions on individual cases. A comparison of this kind is important when it comes to using AI as an assistive technology for clinical decision-making, as it can determine whether or not AI provides significant incremental utility beyond existing tools. AI algorithms can maximize and cooperate synergistically with human assessment by complementing and augmenting human decisions. On the other hand, clinicians would have little interest in or benefit from incorporating AI algorithms if their decisions –and errors– highly overlap with their own. We aim to address this issue by examining whether or not AI detects diagnostic indicators that may go unnoticed by human observation.

In this paper, our main contribution is comparing the performance of AI and humans with knowledge of autism in accurately classifying autism from a 3-minute get-to-know-you conversation with a non-clinician conversation partner. Specifically, we implemented a computer vision pipeline for predicting autism using features of facial behavior during conversations with a sample of *N* = 42 adults − 15 individuals with autism spectrum disorder (ASD) and 27 neurotypical (NT) individuals. We then recruited a total of 19 human raters (8 expert clinicians, 11 non-experts with experience with autism) to predict the diagnostic status of the same participants. The expert raters were doctoral level clinicians with extensive training on autism, while most of the non-experts were BA level researchers still learning about autism. Raters watched the same videos of participants’ faces during conversations that were fed to the computer vision pipeline, without sound to allow for a fairer comparison with the AI algorithm.

Results suggest that the AI pipeline based on participant facial behavior predicts diagnostic status with 80.5% accuracy. This accuracy was comparable to the 80.3% overall accuracy achieved by human raters (83.1% for experts and 78.3% for non-experts), demonstrating the potential of AI to detect facial behavioral patterns that differentiate adults with autism from neurotypical peers in the context of a casual, get-to-know-you conversation. Moreover, we show that the prediction errors of AI and humans had little overlap, indicating that the AI can provide complementary information that could prompt and assist clinicians with their evaluations and decision-making. The fact that all the results of this paper are extracted from a brief naturalistic conversation is a significant contribution, as a 3-minute conversation with a non-expert is a highly scalable paradigm, and thus a promising option as a screening or (preliminary) diagnostic procedure. The results of this paper motivate further research efforts to understand the decision mechanisms of AI algorithms, particularly for uncovering subtle behavioral patterns in psychiatric conditions.

## 2. Participants and Procedure

Forty-four adults participated in the present study (ASD: n=17, NT: n=27, all native and fluent English speakers). Participant groups did not differ significantly on mean chronological age, full-scale IQ estimates (WASI-II) [9], verbal IQ estimates, or sex ratio (Table 1). Participant diagnostic status (ASD or NT) was confirmed as part of this study using the Clinical Best Estimate process [10], informed by the Autism Diagnostic Observation Schedule - 2nd Edition, Module 4 (ADOS-2) [11] and adhering to DSM-V criteria for ASD [12]. All aspects of the study were approved by the Institutional Review Board The Children’s Hospital of Philadelphia (CHOP). Two participants were excluded from analysis due to their lack of consent for this particular set of experiments or their data being unavailable for processing, yielding a final sample of 42 participants (ASD: N=15, NT: N=27).

Participants underwent a battery of tasks that assessed social communication competence, including a slightly modified version of the Contextual Assessment of Social Skills (CASS) [13]. The CASS is a semi-structured assessment of conversational ability designed to mimic real-life first-time encounters. Participants engaged in two 3-minute face-to-face conversations with two different confederates (research staff, blind to participant diagnostic status and unaware of the dependent variables of interest). In the first conversation (interested condition), the confederate demonstrates social interest by engaging both verbally and non-verbally in the conversation. In the second conversation (bored condition), the confederate indicates boredom and disengagement both verbally (*e.g.,* one-word answers, limited follow-up questions) and physically (*e.g.,* neutral affect, limited eye-contact and gestures). All analyses throughout this paper are based on the interested condition only.

**Figure 1:**
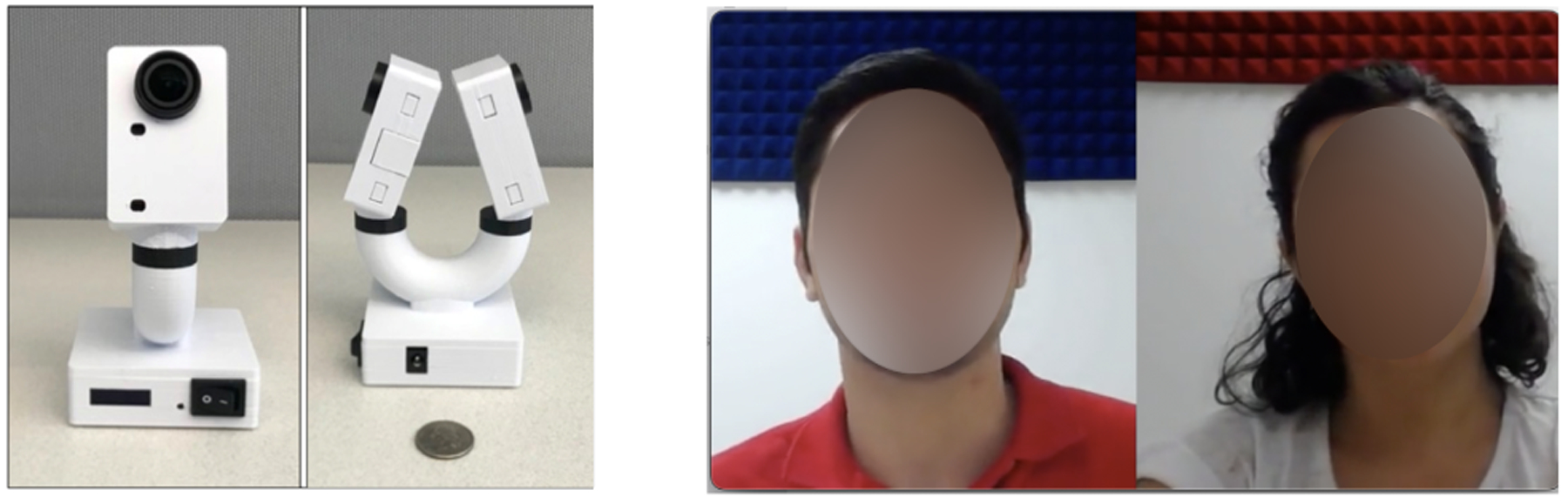
Left: The device used to record the conversation. The device has two cameras, each pointing to one party of the conversation. Right: Example of videos shown to the human raters. The video contains synchronized videos of the heads/faces of both the participant and the confederate as recorded by the device on the left. The video of the participant’s face only served as input to the AI pipeline.

During the CASS, participants and confederates were seated facing one another. Audio and video of the CASS were recorded using an in-house device comprising two 1080p HD (30 fps) cameras (Fig. 1), which was placed between the participant and confederate on a floor stand. The two cameras of the device point in opposite directions to allow simultaneous recording of the participant and the confederate. However, the AI analyses in this paper are conducted on the video data of the participant only. In other words, even if the context of the conversation is *dyadic*, our AI-based analysis is not dyadic since it discards the information from the confederate and focuses only on the participant. We refer to this type of analysis as *monadic* analysis.

CASS confederates included 10 undergraduate students or BA-level research assistants (3 males, 7 females, all native English speakers). Confederates were semi-randomly selected, based on availability and clinical judgment. In order to provide opportunities for participants to initiate and develop the conversation, confederates were trained to speak for no more than 50% of the time and to wait 10s to initiate the conversation. If conversational pauses occurred, confederates were trained to wait 5s before re-initiating the conversation. Otherwise, confederates were told to simply naturally engage in the conversation. Prior to each conversation, study staff provided the following prompt to the participants and confederates before leaving the room: “Thank you both so much for coming in today. Right now, you will have 3 minutes to talk and get to know each other, and then I will come back into the room.”

## 3. Prediction of Autism Diagnosis

### 3.1. Human Raters

We recruited a total of 19 human raters to view the videos from the sample of *N* = 42 participants. Eight of the raters were autism clinical experts, doctoral level clinicians with extensive training at the Center for Autism Research (CAR) of CHOP. The remaining 11 (non-expert) raters had some familiarity with autism but not specialized training and worked at CAR. Most of these non-expert raters were BA-level psychology students learning about autism.

The videos that were shown to the human raters were prepared as follows: First, we cropped the videos of the participant and their corresponding confederate conversation partner so that only the heads and necks were visible. Next, we combined the synchronized videos of the heads/faces of the participant and confederate into a single video file per participant such that participant and confederate were positioned side by side (Fig. 1, right). The audio was removed in order to allow human raters to focus on the facial behavior, as was the case for the AI algorithm. The videos for all *N* = 42 participants were presented to human raters in a random order on high resolution monitors.

Raters were instructed to watch each video just once and to make a decision as to whether the study participant had autism or not. They were told that all participants were either confirmed to have autism through clinical evaluation by a licensed expert, or were recruited specifically as neurotypical controls (*i.e.,* clear cases of individuals without autism). Raters were not allowed to go back and review earlier videos. They were instructed to watch all videos within 1 to 3 viewing sessions, with nearly all being completed in 1 or 2 sessions.

### 3.2. Computer vision

#### 3.2.1. Quantification of facial behavior

Our goal is to quantify all observable facial behavior of a participant, which includes facial expressions and head movements. Also, we did not want to limit analysis to emotion-related expressions (*e.g.,* the six basic emotions), as other kinds of facial movements (*e.g.,* communicative expressions, speech-related mouth movements) are also important for diagnosing autism [14]. Therefore, we quantify behavior using a 3D morphable model (3DMM) [15] as 3DMMs contain expression bases (*e.g.,* [16]) that can quantify any facial movement. Moreover, 3DMMs can simultaneously model facial identity, pose, and expression. This increases the precision of parsing facial expressions and head movements, since the effect of identity (*i.e.,* identity bias [17]) is reduced when modeled and thus explained away. Specifically, we use the 3DI method [18], as it can learn identity from multiple frames and thus model and remove its effect more accurately. Moreover, 3DI can take the parameters of the camera as input, which is critical for increasing the accuracy with which facial expressions and pose are decoupled [19].

**Figure 2:**
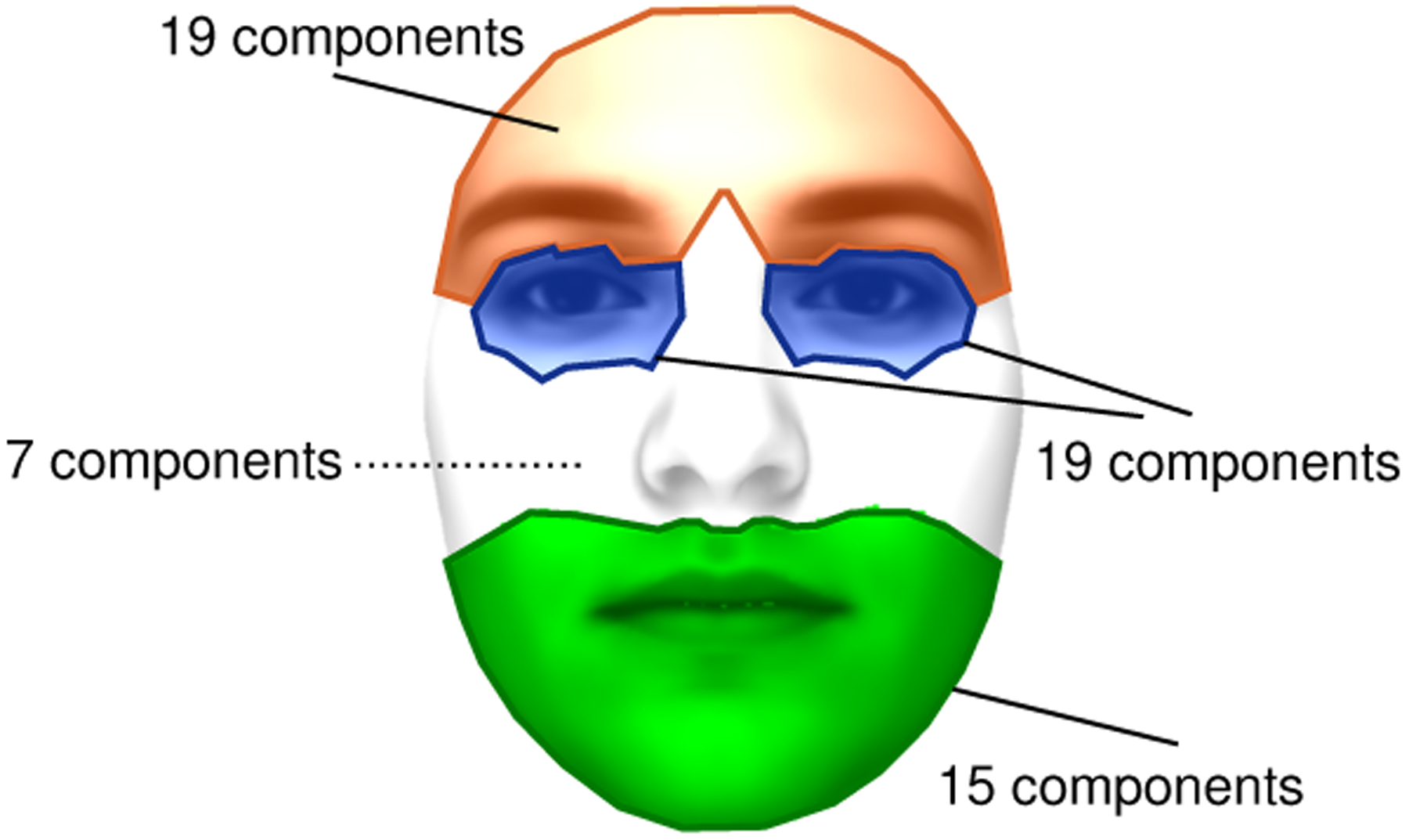
We divide the facial mesh of *P* points into the four groups illustrated in this figure: (1) brows and forehead; (2) eyes; (3) nose and cheeks; and (4) mouth and chin. Each of the *P* mesh points is assigned to one of these four groups by first computing the distance of the point to all the 51 facial landmarks (iBUG-51 [19]), and then identifying the facial feature (*i.e.,* brow, eye, nose or mouth) corresponding to the closest landmark. The expression basis that we use has a total of 60 components, distributed as shown in the figure.

A 3DMM method produces a dense mesh of *P* three-dimensional points **X** ∈ ℝ^3×P^ to represent the face in a given video frame **I**. (*P* is 23, 660 for the 3DI method). This 3D mesh is a function of the facial pose (*i.e.,* a rotation matrix **R** ∈ ℝ^3×3^ and a translation vector ***τ*** ∈ ℝ^3×1^), the facial identity of the person $\bar{\text{X}}$ and the facial expression variation in the image **ΔX** ∈ ℝ^3×*P*^ :

(1) $$\text{X}=\text{R}(\bar{\text{X}}+\Delta \text{X})+\text{T},$$

where the columns of the matrix **T** ∈ ℝ^3×P^ are identically ***τ***. The matrices of interest in the scope of our study are the matrix of head rotation **R** and the expression variation, **ΔX**. 3DMMs represent expression variation as a linear sum, **ΔX** = **WƐ**, where *Ɛ* ∈ ℝ^*K*×1^ is the vector representing the expression. The expression basis **W** used by 3DI method is constructed via PCA [16], which limits the interpretability as PCA components are not localized–we cannot associate any PCA component with a specific facial region. To make the results of our study more interpretable, we modified the expression model in a way that the resultant expression model, **W**^***′***^, contains 60 localized basis components as shown in Fig. 2. Using this model, we represent the expression variation in the image with the vector *Ɛ*^*′*^ that minimizes the norm *||***ΔX** − **W**^***′***^*Ɛ*^*′*^*||*_2_. We ignore the 7 components that correspond to the nose and cheek regions (Fig. 2), and we finally represent the expression variation in a video of *T* frames with a matrix **E** of size *T* × 53, obtained by horizontally concatenating the expression vectors from all the frames. Finally, using the rotation matrix **R** estimated at each frame, we compute the yaw, pitch and roll angles per frame, and represent head rotation throughout the video with a matrix **Φ** of size 3 × *T*. The facial movement variation and head rotation of a person throughout the video are represented together with a matrix **Y** of size 56 × *T*, obtained as

(2) $$Y\text{=}\left[\begin{array}{c} E\\ \Phi \end{array}\right].$$

Alternatively, one can consider using the Action Units (AUs) of the Facial Action Coding System instead of the 3DMM-based expression features that we used above. However, our analysis is based on correlation of time series (Section 3.2.2), which requires a representation where AU intensity needs to be provided—binary AU labels would be very limiting. Since automated AU detection systems (*e.g.,* OpenFace [20]) provide AU intensity only for a relatively small number of AUs, we preferred to use the 3DMM-based features instead of the AUs. One could also consider to add the AU features to the features **Y** above, but we refrained from doing so, because the number of our correlation features increases exponentially with the number of rows in **Y** (Section 3.2.2). This also explains why we refrained from adding the features from nose and cheek regions, as the potential extra information that would be provided by these regions may not justify the exponential increase in the dimensionality of the feature space. That said, the utility of all such extra information should be explored in future AI pipelines that can be trained with data from larger samples.

#### 3.2.2. Correlation features

An important aspect of social communication is how different modalities of communicative behavior are integrated and coordinated. For example, the ADOS, the gold standard clinical assessment for autism diagnosis, includes criteria that evaluate how an individual combines speech with gestures and eye contact with facial expression [14]. Similarly, the coordination of behavior within a communicative modality (*e.g.,* movements across different parts of the face) is important; for example, atypical aspects of facial expressions can be characteristic of autism [21, 22]. Thus, to capture coordination across different types of facial and head movements within a person, we apply windowed cross-correlation [23] on the matrix **Y**. That is, considering the *i*th and *j*th row of **Y** as two time series, we compute the cross correlation between the two, over time windows of length *T*_*w*_ and a step size of *T*_*w*_*/*2 (*i.e.,* consecutive time windows have an overlap of 50%). We then compute the average *μ*_*i*_,_*j*_ and standard deviation *σ*_*i*_,_*j*_ of the maximal cross-correlation values (*w.r.t*. lag) per window. To distinguish between the cases where, say, a mouth movement was followed with a pose variation from the opposite direction, we allow only forward lag on the second time series in the pair, thus (*μ*_*i*_,_*j*_, *σ*_*j*_,_*i*_) is in general different from (*μ*_*j*_,_*I*_, *σ*_*j*_,_*i*_). In sum, since **Y** has 56 rows, we have 56 × 56 ordered pairs, and with 2 features (*i.e.,* mean and standard deviation) per pair, the total number of features that represent the behavior of a participant is *M* = 6272.

#### 3.2.3. Classification

We predict the diagnostic group of participants (ASD vs. NT) using a linear SVM classifier by simply using the default *C* value for SVM (*i.e., C* = 1). We report results based on nested cross-validation, where the only hyper-parameter that is being optimized is the time window *T*_*w*_, and we optimize over values of *T*_*w*_ = 1, 2, 4, 6 seconds. The time window length that was selected in most cross validation folds was *T*_*w*_ = 2*s*.

While more advanced AI models based on deep learning could be used, the sample size is insufficient for reliably training deep learning models from scratch. Moreover, to our knowledge, there is no publicly available pre-trained deep learning model that is directly applicable for our problem, thus taking an existing model and re-training only a part of it (*e.g.,* the classification layer) with our data is also not an approach within reach.

## 4. Results and Discussion

Table 2 shows the prediction accuracy of the human raters and the AI method. The results for the AI method are obtained via 10-fold cross validation (repeated 100 times with shuffling participant order). The average accuracy of expert clinicians is slightly higher than that of non-experts. Of note, the average accuracy of all human raters (expert and non-expert) is similar to that of the AI approach. The average positive predictive value, negative predictive value, sensitivity and specificity of the AI model are respectively 0.86, 0.79, 0.55, 0.95.

We next investigate whether the errors of the human raters coincide with the errors of the AI algorithm. Table 3 shows the participants whose diagnoses were inaccurately predicted by most human raters (*i.e.,* average prediction accuracy < 50%), along with the correct diagnosis and diagnosis predicted by AI. Results show that four out of these five human mispredictions were correctly predicted by the AI, including the first participant in the list, whose diagnosis was predicted correctly by only 21% of the human raters. In other words, participants that were difficult for most human raters to accurately classify were not particularly difficult for the AI. This suggests that the decision mechanism of AI is different than that of the humans, and the following results further support this point of view.

**Figure 3:**
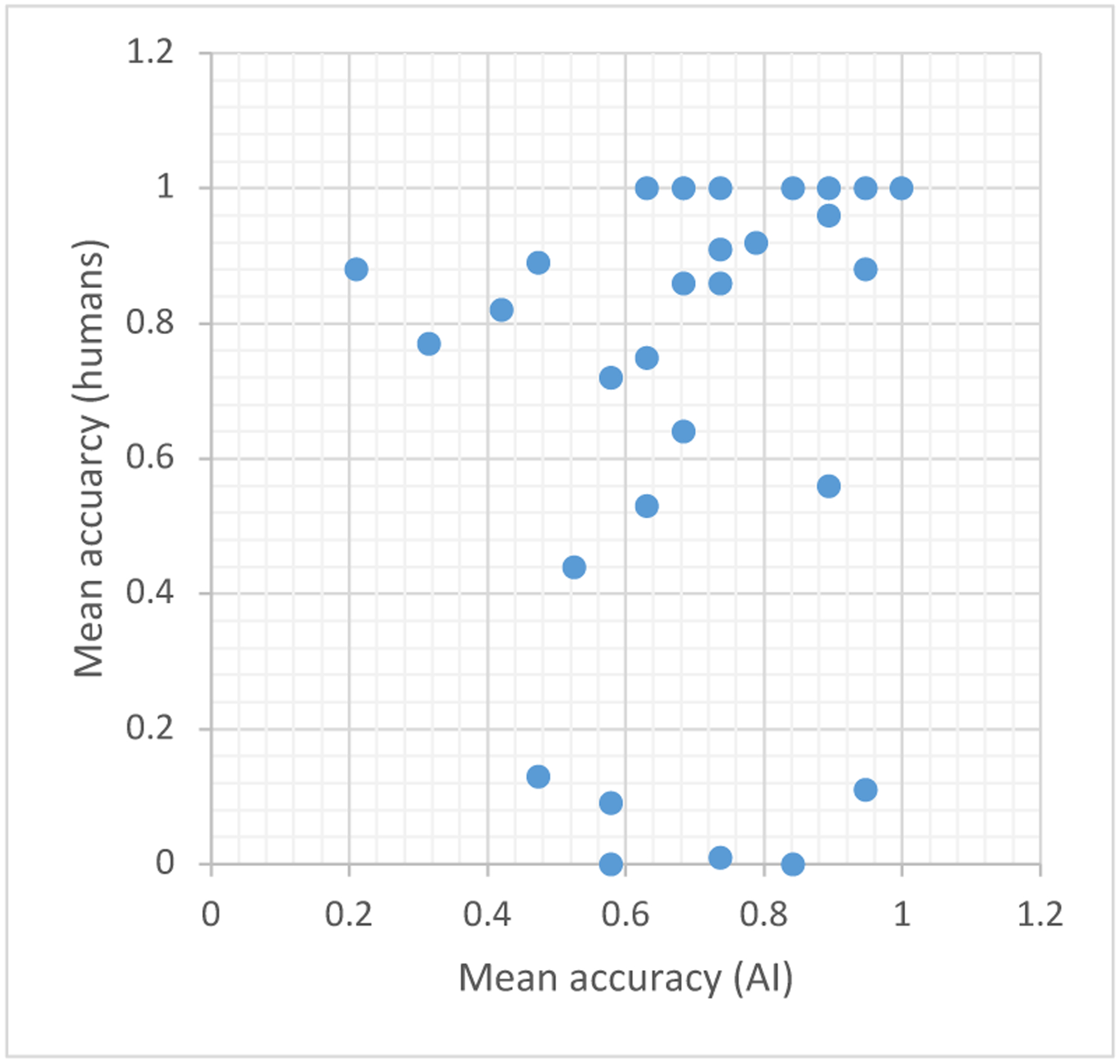
The average prediction accuracy of human raters against the average prediction accuracy of the AI pipeline, per participant. The average prediction for the AI results in this figure are computed by repeating 5-fold cross-validation 1000 times, and averaging over the predicted 1000 predictions per participant.

Fig. 3 plots the average prediction accuracy of human raters against the average accuracy of the AI algorithm per participant. The correlation between these quantities is not strong (*ρ* = 0.35) and is mostly driven by the participants that are correctly classified by both humans and the AI (*i.e.,* the top right points of the plot). For example, if we remove the subjects that are correctly classified by at least 95% of the human raters, the correlation drops to *ρ* = 0.19. The lack of points in the lower-left quadrant of the Fig. 3 supports the conclusion that the diagnoses that were difficult to predict for humans were not typically difficult for the AI, and vice versa.

This outcome further supports that the decision mechanism of the AI is different than that of the humans, and is a desirable outcome if AI is to be used as an assistive technology for human clinical decision-making, since it implies that human decisions can be augmented with the help of AI. For example, in a potential application for autism screening from similar short social videos, humans and AI could simultaneously make predictions, and humans could re-evaluate their decision if it is inconsistent with the decision of the AI algorithm. However, arguably, a scenario of this kind is conceivable only if the AI algorithm produces a semantically interpretable output—that is, the algorithm lists the detected behavioral patterns that lead to a diagnostic decision of autism vs. NT. Otherwise, without any explanation of the prediction, it would be difficult for a clinician to determine to what degree the result of the AI algorithm should be taken into account.

**Figure 4:**
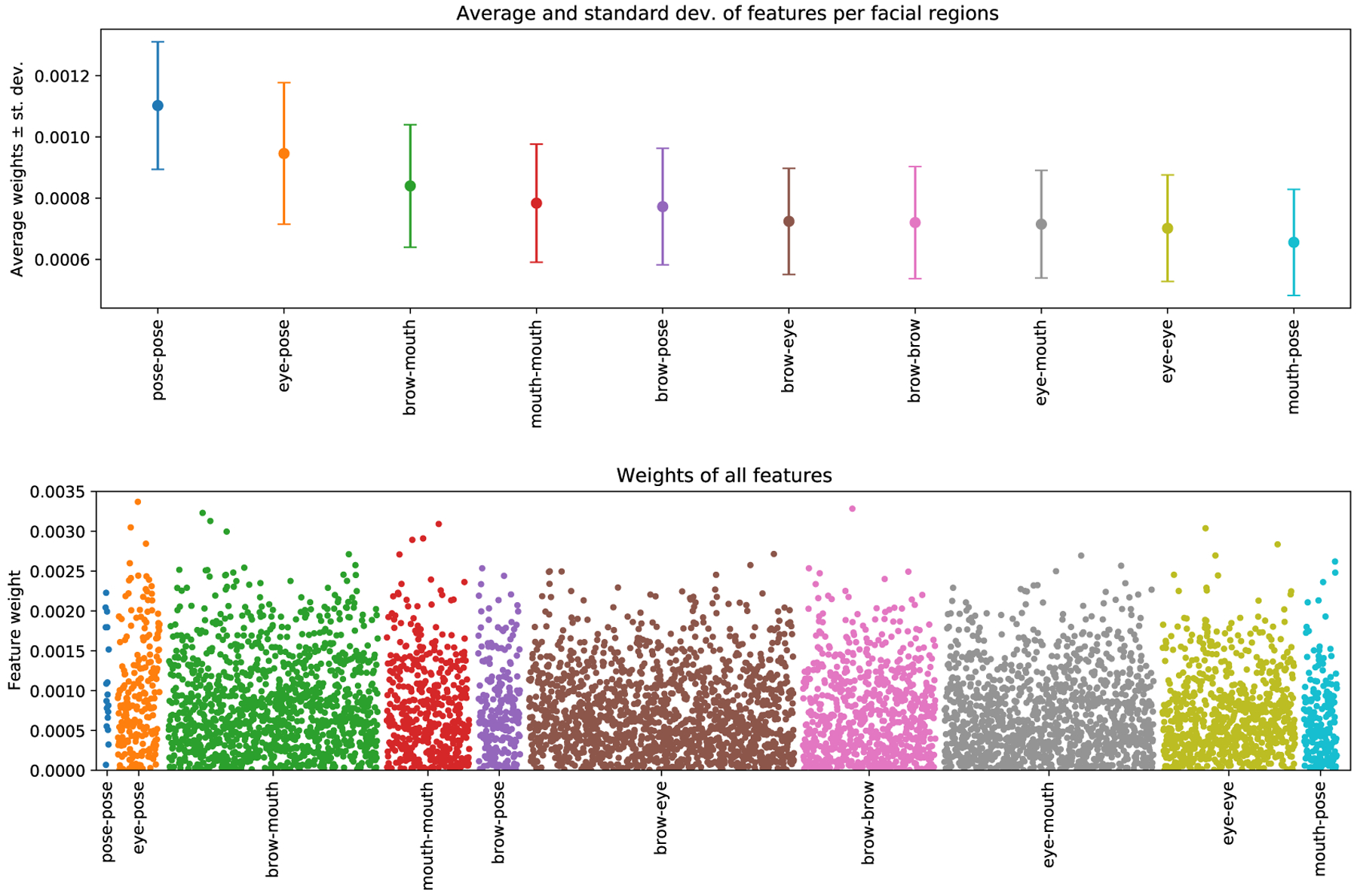
Top: Average and standard deviation of correlation features per facial region; e.g., statistics for eye-pose show are computed from correlation features that are extracted from these two regions (Section 3.2.2). Bottom: Manhattan plot showing all correlation features.

**Figure 5:**
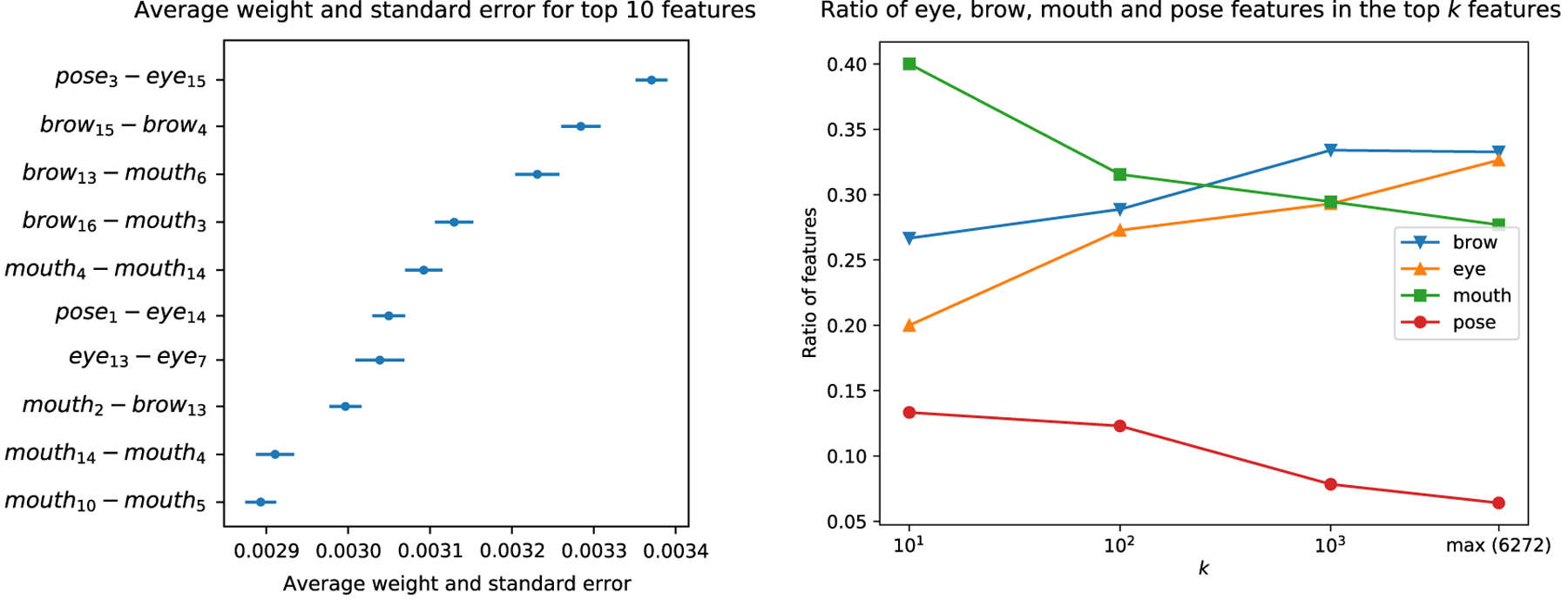
(a) The labels and weights of the top 10 features along with the standard error (across cross-validation folds). (b) The ratio of each of the four feature categories (brow, eye, mouth, pose) in the top *k* features (*i.e., k* features with the highest average SVM weight) against *k*. The graphs are computed on the basis of a feature category appearing on either side of a correlation feature. For example, if a correlation feature is extracted from the correlation between a mouth and a pose feature, it is considered to be both a mouth and a pose feature. The rightmost value of each graph shows the baseline rate for each feature category –the ratio of the feature category in the entire set of 6272 features– highlighting the importance of the mouth and pose features, since they appear more frequently in the top-10, top-100, top-1000 features compared to their baseline rate.

In order to shed some light on the decision mechanism of the AI, we analyze the features that were dominant in the SVM classifier—the features that had greater weight. Fig. 4 shows the weights of all the features and Fig. 5a shows the 10 features that had the greatest (absolute) weight across cross-validation folds along with their names. While a complete analysis of the semantic interpretation of each feature is a difficult task, we can still gain some insight into the SVM decisions by inspecting these results. First, note that pose-pose features (*i.e.,* features that summarize correlation between two head rotation angles) have the greatest weight on average (Fig. 4 top), indicating that head movements are important for distinguishing behavioral patterns of autistic vs NT participants. Moreover, correlation features combining the pose and eye emerge as important both in Fig. 4 and in Fig. 5a, supporting previous literature suggesting that blinking and nodding are important non-verbal behaviors in conversations [24], and head and eye movements are indicators of social attention [25]. Second, mouth-related features also emerged as important. For example, six out of 10 correlation features in Fig. 5a are related to mouth, with three of them being pairs of mouth-mouth features.

We next analyze which, if any, of the four feature categories (eyes, brows, mouth, pose) have greater presence among the top *k* features. Fig. 5b plots the proportion of the eye-, brow-, mouth- and pose-related features in the top-10, top-100, top-1000 most important features, as well as their proportion in the entire pool of 6272 features. For example, while the baseline rate of pose features is only *~*5.3% (*i.e., ~*5.3% of the entire set of 6272 features are pose-related), we see that the top 10 features contain a pose-related feature at a ratio of ~13.3% (see caption of Fig. 5 for the computation pose-related features), indicating that the pose features have ~2.5 times more presence in the top-10 features compared to their baseline. Similarly, the baseline rate of mouth-related features is *~*25.5%, but *~*40% of the top-10 features are related to the mouth, indicating that mouth features also have greater representation in the set of important features compared to their baseline. In sum, our analyses suggest that the AI algorithm places high emphasis on pose- and mouth-related features when classifying between autism and NT groups. Further analysis to uncover why these features are important is beyond the scope of this study, as this would require more granular expression models (*e.g.,* 3D versions of localized bases [26]), because the approach that we designed from an existing model does not allow us to pinpoint the facial movements of interest beyond the level of the partitioned regions in Fig. 2; for example, we cannot distinguish between parts of the mouth, such as upper lip or mouth corner. Still, our analyses allowed a degree of interpretation that corroborates previous findings on the importance of mouth-related movements [2, 4], as well as the central role that head movements have in social orienting, attention and backchannel feedback (*e.g.,* nodding) [27, 28, 24, 25, 29].

## 5. Conclusions and Future Work

In this paper, we studied the prediction of autism from facial movement behavior during casual conversations. Specifically, we compared the predictive accuracy of expert and non-expert human raters with that of an AI algorithm. Results show that, while both humans and the AI are capable of distinguishing individuals with autism spectrum disorder (ASD) from neurotypical (NT) individuals with high accuracy, their errors do not overlap, suggesting that the decision mechanism of an AI algorithm may be different than that of a human. Thus, AI technologies have the potential to provide complementary information to a clinician and become an assistive tool for decision making. Arguably, the most immediate application based on our results is a new, semi-automatic screening technology for autism, where an individual is advised for further diagnostic evaluation in the event that a (non-expert) human or the AI model predicts that the individual exhibits autism-specific behavior. However, in a real life scenario, the problem of interest would be more difficult as a potential patient may not be NT but may not have ASD either. Thus, future research is needed to identify the performance of humans and AI models in predicting ASD diagnosis from neurodiverse samples.

Our results directly motivate further future research in multiple directions. The most pressing future direction from the perspective of making AI an effective assistive tool is examination of the behaviors that lead to a predicted diagnosis. Having interpretable outputs is necessary for using AI technologies in clinics, as clinicians should understand how the AI algorithm makes a prediction before taking this prediction into account. Furthermore, research on younger participants is needed, given that early diagnosis improves access to effective early interventions and thus can improve developmental outcomes. Another future direction is to investigate the benefits of dyadic analysis, where, unlike our monadic analysis (Section 2), the behavior of confederate is also taken to account. Finally, user research is necessary to test if and to what degree clinician diagnoses can be improved through the use of AI assistive tools.

## Figures and Tables

**Table 1:** Participant characterization within our sample. Wilcoxon rank sum tests with continuity correction were used for statistical group comparisons, except for sex ratio where the Chi-squared test was used. One NT participant had missing ADOS-2 scores. RRB=Repetitive Behaviors and Restricted Interests subscore of the ADOS-2.

Variable	ASD Mean (SD)	NT Mean (SD)	Statistics	*p*-value
Age (years)	26.9 (7.3)	28.1 (8.4)	W = 234	0.923
Sex (Male, Female)	15m, 2f	23m, 4f	χ^2^: 0.08	0.774
Full-Scale IQ	102.1 (19.8)	111.7 (9.5)	W = 157	0.080
Verbal IQ	112.6 (22.1)	112.4(11.2)	W = 215	0.736
ADOS Total	13.1 (3.0)	1.1 (0.9)	W = 442	< 2e-8*
ADOS Social Affect	9.8 (2.3)	1.0 (0.9)	W = 442	< 1e-8*
ADOS RRB	3.3 (1.5)	0.1 (0.3)	W = 441	< 1e-9*

* Statistically significant difference between diagnostic groups, *p<*0.05.

**Table 2:** Average prediction accuracy of all human raters, non-expert raters, expert raters and AI.

All human raters	Non expert raters	Expert raters	Al
80.3%	78.3%	83.1 *%*	80.5%

**Table 3:** The five participants whose diagnosis (dx) was mispredicted by most human raters (*i.e.,* average prediction accuracy < 50%), with the corresponding average accuracy by the AI (computed by repeating 5-fold cross-validation 1000 times) and the diagnosis predicted by AI via leave-one-out cross-validation.

dx	Average accuracy (humans)	Average accuracy (AI)	predicted dx (AI, leave-one-out CV)
ASD	21.1%	88.1%	ASD
ASD	31.6%	77.4%	ASD
ASD	42.1%	82.1%	ASD
ASD	47.4%	89.4%	ASD
ASD	47.4%	12.8%	NT
